# Why Don't We Ask? A Complementary Method for Assessing the Status of Great Apes

**DOI:** 10.1371/journal.pone.0018008

**Published:** 2011-03-31

**Authors:** Erik Meijaard, Kerrie Mengersen, Damayanti Buchori, Anton Nurcahyo, Marc Ancrenaz, Serge Wich, Sri Suci Utami Atmoko, Albertus Tjiu, Didik Prasetyo, Nardiyono, Yokyok Hadiprakarsa, Lenny Christy, Jessie Wells, Guillaume Albar, Andrew J. Marshall

**Affiliations:** 1 People and Nature Consulting International, Bali, Indonesia; 2 School for Archaeology and Palaeoanthropology, Australian National University, Canberra, Australia; 3 School of Mathematical Sciences, Queensland University of Technology, Brisbane, Australia; 4 The Nature Conservancy – Indonesia Program, Jakarta, Indonesia; 5 Borneo Orangutan Survival Foundation, Orangutan Reintroduction Program Central Kalimantan, Palangka Raya, Indonesia; 6 Kinabatangan Orang-utan Conservation Project, Sandakan, Sabah, Malaysia; 7 Sumatran Orangutan Conservation Program (PanEco-YEL), Medan, Indonesia; 8 Anthropological Institute and Museum, University of Zurich, Zurich, Switzerland; 9 Fakultas Biologi, Universitas Nasional, Pasar Minggu, Jakarta, Indonesia; 10 Perhimpunan Ahli dan Pemerhati Primata Indonesia (PERHAPPI), Bogor, Indonesia; 11 World Wide Fund for Nature Indonesia, Pontianak, Indonesia; 12 School of Biological Sciences, University of Queensland, St Lucia, Australia; 13 Société d'Ornithologie de Polynésie, Taravao, Tahiti, French Polynesia; 14 Graduate Group in Ecology, and Animal Behavior Graduate Group, Department of Anthropology, University of California Davis, Davis, California, United States of America; Zoological Society of London, United Kingdom

## Abstract

Species conservation is difficult. Threats to species are typically high and immediate. Effective solutions for counteracting these threats, however, require synthesis of high quality evidence, appropriately targeted activities, typically costly implementation, and rapid re-evaluation and adaptation. Conservation management can be ineffective if there is insufficient understanding of the complex ecological, political, socio-cultural, and economic factors that underlie conservation threats. When information about these factors is incomplete, conservation managers may be unaware of the most urgent threats or unable to envision all consequences of potential management strategies. Conservation research aims to address the gap between what is known and what knowledge is needed for effective conservation. Such research, however, generally addresses a subset of the factors that underlie conservation threats, producing a limited, simplistic, and often biased view of complex, real world situations. A combination of approaches is required to provide the complete picture necessary to engage in effective conservation. Orangutan conservation (*Pongo* spp.) offers an example: standard conservation assessments employ survey methods that focus on ecological variables, but do not usually address the socio-cultural factors that underlie threats. Here, we evaluate a complementary survey method based on interviews of nearly 7,000 people in 687 villages in Kalimantan, Indonesia. We address areas of potential methodological weakness in such surveys, including sampling and questionnaire design, respondent biases, statistical analyses, and sensitivity of resultant inferences. We show that interview-based surveys can provide cost-effective and statistically robust methods to better understand poorly known populations of species that are relatively easily identified by local people. Such surveys provide reasonably reliable estimates of relative presence and relative encounter rates of such species, as well as quantifying the main factors that threaten them. We recommend more extensive use of carefully designed and implemented interview surveys, in conjunction with more traditional field methods.

## Introduction

Conservationists widely claim that accurate population estimates are needed for assessing a species' vulnerability to extinction, monitoring population status, and informing decisions about how to best allocate limited conservation funds [1], [2], [3]. Consequently, a great deal of conservation effort and funding is invested in wildlife surveys to estimate population densities. However, many of these surveys, at least in Indonesia, are of limited practical value to conservation practitioners because their estimates are rarely integrated with information on social or economic factors that may be critical to understanding of the causal relationships between species' densities and conservation interventions and threats [4], [5]. Also, field scientists often seek to isolate the effects of one or few factors, by estimating population density in sites representing differences in a single factor, e.g., sites with or without timber harvest. Effective conservation management, however, requires us to deal with complex relationships among many factors. For example, presence of a species in a region may be related not only to timber harvest, but also to other ecological, social, cultural, economic and political factors. Of course, understanding this interplay is important not only for the conservationist, but also for industry (e.g., the manager of the timber concession), government and the people who live in the region.

Such multifaceted problems are difficult to solve, because they require different academic backgrounds and approaches, as well as significant research efforts. Moreover, methods for optimizing multi-objective management plans often do not lead to a single best solution, but to a range of different options, and must be followed by skilled decision making based on high quality evidence about the multiple factors involved. Given methodological limitations and restricted time and funding, conservation researchers often choose to focus on a single aspect of the problem, and thereby fail to develop practical solutions of broader applicability [4]. Here we consider whether this trade-off between multi-facetted conservation needs and the resource requirements to address those needs can be reduced by including structured interviews into a broader sampling framework that also includes field surveys.

Orangutan (*Pongo* spp.) conservation provides a good example of this issue. Despite nearly five decades of orangutan research, practical conservation solutions are still limited. Research has largely been focused on ecological aspects of the species through field surveys in protected areas, where conservation concerns were relatively limited. This has clarified many ecological aspects of orangutan conservation but very few social ones [6]. Orangutan surveys are labour- and time intensive [7], and the rare and cryptic nature of these apes makes surveys based on direct sightings of such animals generally unfeasible and inaccurate, resulting in relatively little information from major efforts. Systematic counts along transects of the resting platforms, or 'nests', that orangutans build have therefore been used as predictors, or as direct proxies, for population density. Unfortunately, variability in nest decay rates means that a survey period of at least 6 months is required in any particular location to obtain accurate density estimates [7], [8], and this limits the effective area that can be reliably surveyed. Even relatively large survey efforts have produced estimates with limited accuracy and precision [9], [10]. These methodological constraints mean that only a small part of the species' range can be surveyed; for example, until 2004, such surveys for orangutans in the Indonesian part of Borneo covered an estimated 20–25% of their total range [11]. This is not a fault of the survey methodology, but it indicates that there is a mismatch between information required for effective conservation, currently employed methods, and resources available for surveys.

Nest surveys address only a subset of the factors that influence orangutan presence and abundance and they typically are unable to assess the magnitude of specific threats or their impacts. For example, only a handful of studies have addressed the impact that selective timber harvest in natural forest has on orangutans [12], [13], [14], [15], [16], and only one of these [15] has tried to identify which particular aspects of timber harvest affect orangutans, and how managers could mitigate the impacts. Socio-cultural factors are also severely under-represented in this body of research. For example, despite several modelling exercises indicating that hunting could be a crucial factor in the orangutan's decline [17], [18], [19], [20], only one published study has specifically assessed the impact of hunting on the species [16]. There is a clear lack of understanding about the attitude of local people to the conservation of orangutans and their habitats, perceived threats and their underlying causes, trade-offs between conservation and economic development, and so on [6]. The lack of such practical, multi-faceted information makes it nearly impossible to determine optimal conservation strategies, and to reconcile orangutan conservation with local aspirations for social and economic development. This may be one of the reasons why so little progress has been made in orangutan conservation over the past few decades [21].

The above suggests that the complex nature of conservation management requires other approaches that can complement current survey methods. One such approach is to interview local people. Interview-based surveys are widespread in other disciplines, but have been used relatively infrequently in conservation assessments [22], [23], [24], [25], [26]. Some of the reluctance to employ such surveys arises from a paucity of examples of their successful implementation, as well as concerns about their rigour [23], in particular possible biases and poor data quality [26], [27], [28], as well as inherent difficulties of collecting data on sensitive topics [29]. As in other disciplines, problems with biases and data quality may arise at any of the three main phases of such a survey. At the design stage, questions can be raised about the sampling frame and sampling scheme (did the people we talked to accurately represent the broader target population?), the questionnaire (were questions well posed or potentially leading?), and the survey preparation (was a pilot study conducted, and how well were interview teams prepared?). At the data acquisition stage, key potential issues relate to data collection (e.g., were the data recorded faithfully?), respondent selection (e.g., did non-respondents differ meaningfully from respondents, or did respondents selected in different ways differ in their responses?), respondent reliability (could they reliably identify the focal species?) and respondent recall [30] (did respondents give consistent replies about an event?). Finally, at the analysis stage, much care must be taken to assess data quality, develop appropriate metrics, deal with missing data, choose appropriate statistical methods and models for estimation and inference, assess the sensitivity of the results and inferences, and produce accurate estimates of population-level variables, based on the sample statistics.

In this paper we evaluate structured interview surveys as a complementary conservation tool. We focus on a recent survey to assess the distribution and threats to the Bornean orangutan (*Pongo pygmaeus*) in Kalimantan, the Indonesian part of the island of Borneo, which involved interviews with nearly 7,000 local people. We discuss the methods we employed to ensure data quality and avoid potential biases, and the implications of our findings for broader application of interview survey methods. Due to the novelty of this survey approach, the present paper discusses methodological issues in depth, and detailed results of our project will be published elsewhere.

## Methods

### Ethics statement

The interview survey approach was reviewed and approved by the Nature Conservancy social science specialist. Participants in the surveys were informed of the goal of the interviews and ensured that the data would be analyzed anonymously (see Text S1).

### (i) Design

#### Sampling frame

The orangutan survey was developed and administered by The Nature Conservancy (TNC) and the Indonesian Association of Primatologists (PERHAPPI), and implemented by 18 local non-government organisations (NGOs). One national and three regional coordinators were hired for training of field teams and technical assistance. The sampling frame encompassed all regions in Kalimantan where orangutans were suspected to occur [21], excluding specific areas (such as national parks) for which data on orangutan distribution, and to some extent threats, already existed. The area hypothesised to encompass the full range of orangutan in Kalimantan was created by taking a 2004 distribution range [17], which was largely based on absence/presence derived from opportunistic interviews in the early 1990s [31] and buffering it with a 5 km zone on its periphery. In total, 1725 villages were identified that occur within this range (West Kalimantan: 558 villages; Central Kalimantan: 976 villages; and East Kalimantan: 183 villages); from among 4200 villages in all of Kalimantan.

#### Sampling scheme

A two-level sampling scheme was employed. The first level comprised a 40% sample of the villages within the orangutan range, stratified by high/medium/low threat of land-use change, based on the allocation of land to plantation and agriculture, mixed landscapes with plantations and nature forests, or natural forest concession or protected forests, respectively. All high threat villages were included; others were selected randomly, resulting in a total of 687 interview villages. The second level comprised selection of ten villagers within each village for interview; this selection process is described below.

The interview surveys were conducted over a period of 15 months from April 2008 to September 2009, and subsequent data analysis and reporting took another 7 months.

#### Questionnaire design

The questionnaire comprised 32 questions and 34 optional sub-questions. These were initially drafted in English (Text S1). Subsequently these were distributed to several orangutan experts for initial review, and again discussed during a one-day workshop in Jakarta involving several Indonesian and international orangutan experts as well as socio-economic survey experts. This process resulted in a list of questions on which all experts agreed. The questions were subsequently translated into Indonesian and given a final review during the provincial training workshops. Despite different ethnic backgrounds, all respondents were found to speak good Indonesian.

#### Preparation

A pilot study was undertaken in two villages in a field site in East Kalimantan. The study allowed identification of problems with the data acquisition equipment, and whether any of the questions were confusing. The results of the pilot study were analyzed, resulting in some minor changes in the final methodology.

Participating NGOs were selected during workshops held in the three provinces in Kalimantan that harbour orangutans. Several training sessions were provided to all potential survey teams to develop a shared understanding of the need to work according to a shared protocol in all parts of the distribution range (and not use different approaches in different areas), and to familiarize the teams with the standardized methods and equipment.

### (ii) Data acquisition

#### Data collection

Digital recording equipment was used as opposed to recording answers on paper, because it standardized the method for all groups and reduced the potential error when transferring data from paper into electronic form. Interview teams were provided with Palmtops equipped with the Episurveyor software (www.datadyne.org/episurveyor). Each team was equipped with a GPS camera which was used to take photos of survey locations and mark them with a latitude and longitude. This method provided evidence that survey teams had indeed been to the survey locations. All teams were provided with an external hard drive on which to back up data whilst in the field and reduce the chance of data loss. Each survey team consisted at a minimum of two people: one survey team leader who would also ask the questions, and one data recorder, GPS and camera operator.

Especially at the start of the surveys, several teams required technical assistance for appropriate interview techniques and data storage and management. Nine teams requested to work on paper rather than use the Palmtops but this was strongly discouraged; still, one team continued to fill in answers on paper before transferring them to a database. No problems occurred with data storage or transfer to the project database, although frequent communication between the coordinators and interview groups was required to ensure a smooth process.

#### Respondent selection

The normal procedure when first entering a village was to go to the house or office of the village head, or if not available, other village leaders. There the team would explain, without use of a standard text, the goal of the interview surveys, its rationale, and their wish to interview 10 people in the village who potentially had knowledge about local wildlife. The team would then ask the village leader for names of qualified informants, and these people were subsequently visited and interviewed. If this first selection round did not deliver 10 people, information about further informants would be requested from the respondents, and, if present in the village, these would then be interviewed. The questionnaire included a question regarding the selection method used for each interviewee. The survey teams were trained to use a standard text at the start of each interview. The text did not mention orangutans.

#### Respondent reliability

The reliability of a villager's responses about orangutans was determined by asking respondents to identify nine mammal species from a set of photographs, including three locally occurring primate species: orangutan, Red Langur (*Presbytis rubicunda*, a primate of similar colour to orangutan), and Bornean Gibbon (*Hylobates* sp.).

Despite careful questionnaire design and testing, a response bias could arise in a number of ways in this survey. For example, respondents could be enthusiastic about answering in a positive manner, or alternatively be reticent about answering questions about orangutan threats, in particular issues regarding hunting, killing and awareness of Indonesian law about protection of the species. Efforts to reduce this form of social desirability bias [29] included assuring participants about the anonymity of their responses, avoiding mention of orangutans in both the introduction to the study and the first section of the survey, and asking about sensitive issues both around the village in general and based on their own personal experiences. A second example of how a bias might arise is through differential recall ability for different time periods. It is well known that events (e.g., sighting of an orangutan) in the last week will be more clearly remembered than similar events in the past year. Such differential recall may lead to inaccurate responses, or alternatively conflicting responses such as reporting that an orangutan was seen in the last week but not in the last month. The questionnaire was structured to ask questions about a range of related issues (e.g., forest trips, orangutan encounters, conflict as indicated by an orangutan entering a village garden, killing or hunting, perceived trends, attitudes to the forest), at a range of locations (e.g., anywhere, around the village), over a range of timeframes (e.g., ever, in the past year), and so enabled some cross-validation of responses. However, this cross-validation is limited in scope and further methods would be required for a full examination of the impacts of recall bias on our inferences.

### (iii) Analysis

The survey generated two main datasets: a ‘village dataset’ comprising characteristics of sampled villages, and a ‘villager dataset’ comprising questionnaire responses from the interviewees.

#### Data quality

Analysis of questions related to orangutans was restricted to respondents who were considered to be able to recognise an orangutan with medium or high reliability. The respondent's reliability was classified as ‘high’ if the respondent correctly identified orangutan, Red Langur and Bornean Gibbon; ‘medium’ if he/she correctly identified orangutan and one of the two other species; and ‘low’ if he/she failed to recognize the orangutan, or only knew the orangutan but neither of the other species. Reliability was also assessed by cross-validation of responses (e.g., ever seeing an orangutan and seeing one in the past year). We had included one species (the Douc Langur from Indochina) to determine false positives, but we could not consistently determine from the answers whether respondents had misidentified the species or considered it absent in the area. We therefore did not use the false positives to determine respondent reliability. We used the reliability assessments in all questions related to orangutans, but for analyses of questions not related to orangutans relevant data from all respondents were used.

#### Metrics

‘Reported Presence’ of orangutan was defined if the respondent reported seeing an orangutan in the past year, and had reported previously seeing an orangutan near the village. The variable was categorized as binary (present/absent).

The survey and questionnaire design precluded direct and absolute measures of abundance or density, and so a relative encounter (RE) measure was derived at both the individual and village level. This was defined as RE  =  reported presence of orangutans in the last year/potential of encountering an orangutan, where potential of encountering an orangutan was calculated using a combination of responses to questions involving the number of trips to forest per year, reason for travel to forest and number of nights spent in the forest on each trip. The number of forest trips over the past year (*FT*), as answered in the interview, was scaled to more clearly differentiate propensity to observe orangutan. The scaling was developed by field experts, and scaled values were as follows: >4 trips/week: *FT* = 260; 2–4 trips/week: *FT* = 156; 1–2 trips /week: *FT* = 78; 1–2 trips /month: *FT* = 18; 1–2 trips /year: *FT* = 2; <1 trips /year: *FT* = 1; and 0 trips: FT = 0. The average trip duration (*Tr D*) was estimated from the number of nights that respondents reported spending in the forest on each trip, using the following rescaling method in which *TrD* is a multiplier of *FT*: if 0 nights then multiply *FT* by 1; if 1–4 nights then multiply *FT* by 1.1; and if 4 nights then multiply *FT* by 1.25. A value of one was assigned to the trip duration if people saw an orangutan but did not enter the forest. The RE figures were computed at both the individual (villager) level (for analysis) and the village level (for mapping). To map the reported presence of orangutans, we calculated the mean RE value for each village, based only on the reliable respondents (i.e., reliability>1). The resulting scores were classed according to quartiles, of zero, low, medium, and high Relative Encounter rates. These values were later compared to abundance or presence-absence estimates from other studies.

#### Missing data

Three datasets were constructed for analysis: (i) consisting only of complete responses; (ii) based on complete responses after inferring missing data in instances where this was obvious (e.g., if the respondents had reported seeing no orangutans in the last year, then a missing observation for ‘how many orangutans have you encountered in the last year?’ can be reasonably inferred to be zero; in contrast, it is less straightforward to infer missing data about the age of a village (see Text S1); (iii) based on complete responses after inferring missing data where this was obvious (as above), and then using multiple imputation to estimate the remaining missing data. The imputation was achieved using ten-fold model-based imputation in the statistical software MLWin 2.22 (Centre for Multilevel Modelling, 2010). Under this approach, a random sample is taken from the original data with replacement and the model is applied to predict (impute) the original missing values, given the observed data [32]. This is repeated ten times. Each missing value is then estimated by the mean of the ten predicted values, with a corresponding variance which reflects the uncertainty of the imputation.

#### Statistical methods

A primary set of target estimates and inferences was constructed prior to examination of the data. A secondary set of estimates and inferences was then constructed based on interesting outcomes of the data analysis. Primary estimates and inferences were based directly on the questionnaire and included orangutan sightings, association with identified threats, perceived trends, attitudes to the forest and so on. Secondary estimates and inferences included sub-analyses generated by the primary analyses, for example disentangling ethnic and religious differences in conflict or perceived trends, and analysing the association between land-use and orangutan presence. In line with statistical practice, it was acknowledged that while the primary set was valid for hypothesis testing, the secondary set was valid only for generation of hypotheses, to be pursued and confirmed in subsequent studies.

The data were analysed in two stages. First, standard exploratory models, hypothesis tests and simple or multiple regression analyses were undertaken to generate sample statistics and compare subgroups based on the primary and secondary sets of hypotheses as described below. Subgroups were defined on the basis of demographics such as age and ethnicity, or questionnaire responses such as reported level of orangutan conflict or attitude to the forest. Second, a multilevel model was employed for the substantive statistical analyses, in which villagers were nested within villages. The results reported here are from the multilevel model, since this model reflected the survey design. Both villagers within villages, and villages themselves, were treated as random effects, so that interest was in accounting for these two sources of variation and obtaining overall estimates (as opposed to estimation of characteristics of each village and villager individually). The multilevel model was applied to the villager dataset alone, and to the villager and village datasets combined.

A number of statistical packages were used for the analyses. These included SPSS and SAS for the summary analyses, and MLWin for the multilevel analyses, which were fit as generalized linear mixed models.

#### Sensitivity analyses

Assessment of the sensitivity (or inversely, the robustness) of results and inferences to data quality, potential biases and statistical assumptions, is a key step in any statistical analysis. In addition to the evaluations described above, two further sensitivity assessments were undertaken to confirm the results and inferences in this study. First, for each primary and secondary analysis, supplementary statistical models were fit, comprising alternative combinations of covariates. Second, the final statistical models were applied to five subsets of the data, each comprising a simple random sample of 80% of the villagers.

#### Orangutan presence and threats

Metrics of orangutan presence, relative encounter rates and threats were obtained at the level of provinces, or across the full Kalimantan range. The process used to extrapolate from sample-based to population-based estimates was as follows. First, the demographics of the respondents based on the questionnaires were compared with relevant general census data for the study area from the Indonesian Bureau of Statistics. For each age and sex category, the ratio of the number of respondents to the relevant population was obtained. Second, the sample estimates were extrapolated to the population in each province by reweighting the estimates based on the sampling ratios. Third, a finite population correction (FPC) factor was employed to adjust the variances of the predicted values, to allow for the fact that a relatively large proportion of the study area was sampled. Note that this approach assumes a random sampling design, but could be adjusted to take into account more elaborate designs if required. It does, however, take into account major demographics, the sampling frame (the study area) and the relative size of the study. Finally, the sample estimates were compared with estimates obtained from other sources where possible.

For this last step, the accuracy of interview-based presence/absence and relative encounter measures was assessed by conducting 48 additional interview surveys in four villages in the Lower Kinabatangan Wildlife Sanctuary in Sabah, Malaysian Borneo, and 58 interviews in five villages around the Lesan protected forest in East Kalimantan [33], and comparing the results to actual densities estimates based on recent nest count surveys. For the Sabah areas, precise information on local orangutan densities was already available from a decade of ground and aerial surveys [14]. For Lesan, orangutan densities had previously been estimated [7], and additional line transect surveys and nest decay studies were conducted at the time of the interviews.

## Results

### (i) Design

#### Sampling frame

**Figure 1:**
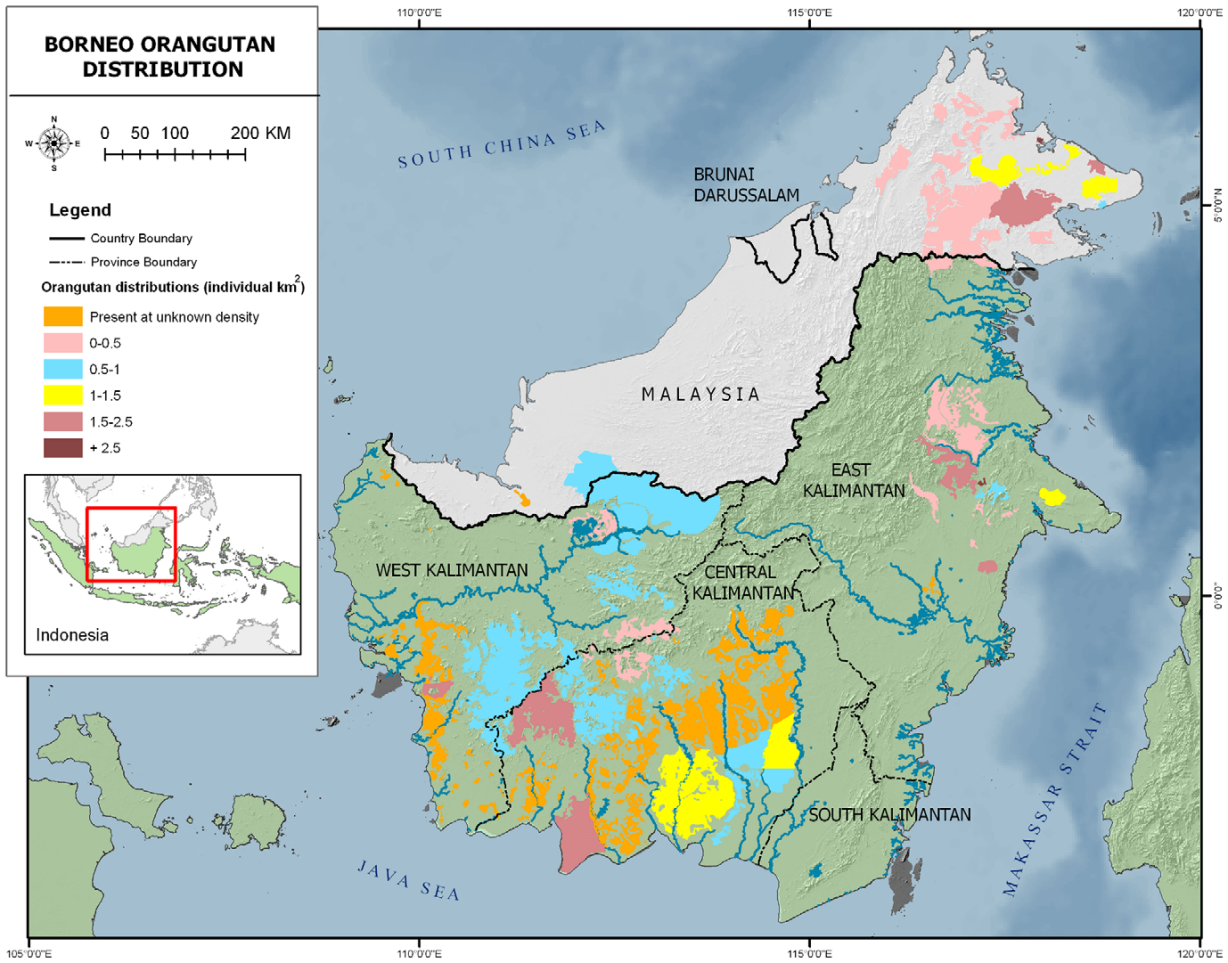
Orangutan distribution and density estimates based on 2003 surveys [**50**]. Actual densities in Kalimantan were known for a few dozen sites and subsequently extrapolated to other parts of the range.

**Figure 2:**
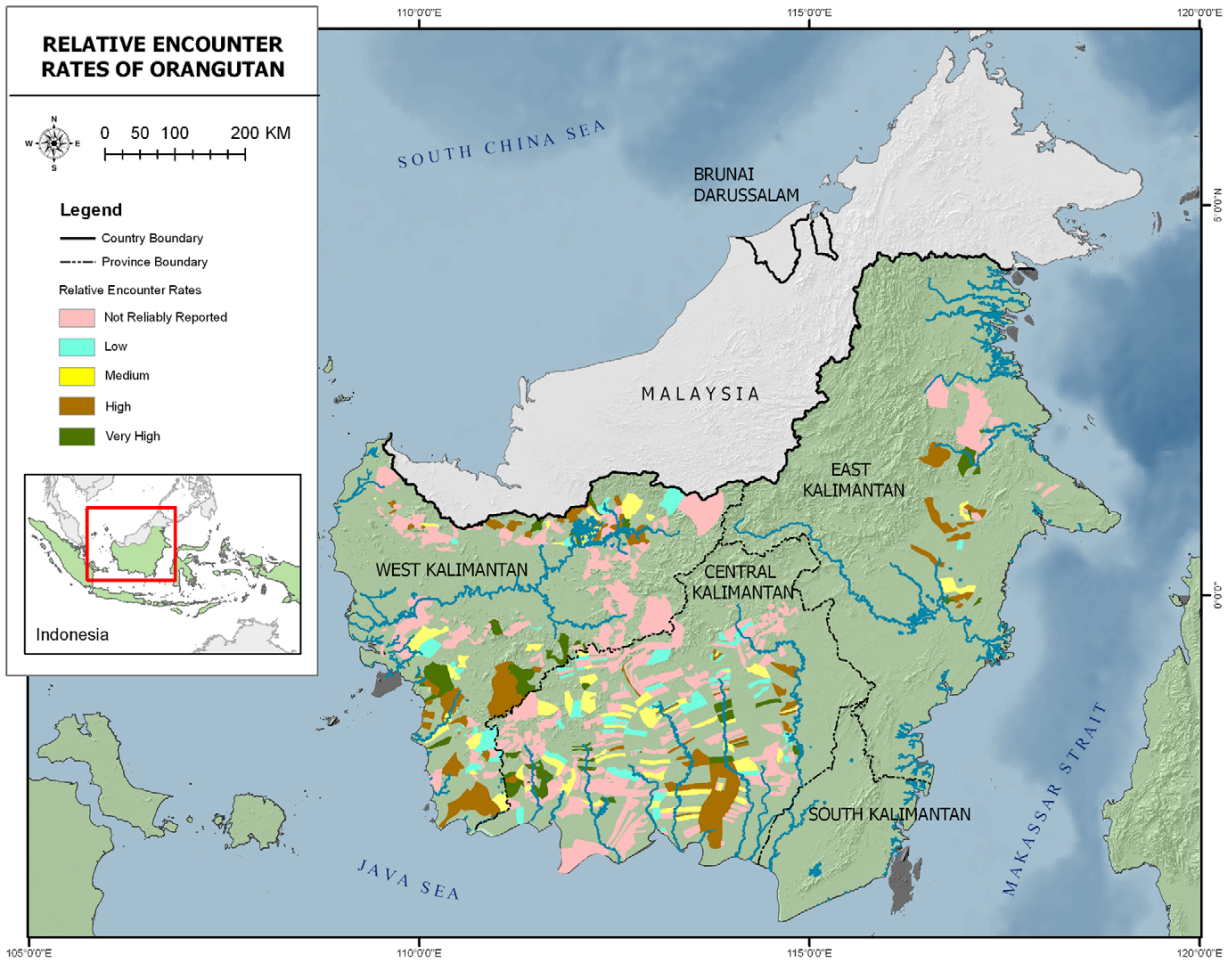
Orangutan encounter rate at village level based on interview surveys. Note that this only included reliable respondents.

The survey yielded reported orangutan presence and relative encounter estimates from a total area of 101,107 km^2^, which is the total administrative area of the 693 villages where we conducted interviews, as mapped by the Indonesian Government. Such village areas include the actual village as well as the surrounding agricultural lands and forests Although the survey did not permit estimation of population density, these results provide an improved and more detailed picture of potential presence (Figure 1) and relative abundance (Figure 2) compared to the previously available map. In several small village areas in northern West Kalimantan where orangutans were thought have become extinct, orangutans were still reported. The same was found in southern East Kalimantan. Additional field surveys are required to determine whether these populations are viable

#### Sampling scheme

A total of 693 villages were included in the sample, six more than initially selected. These additional villages were selected by the survey teams when they were in the field. All but one of the originally selected villages were surveyed, and all villages where surveys were attempted yielded respondents. One village had been abandoned and the survey team substituted this village with the new, nearby village to which the villagers had moved.

A total of 6972 villagers were interviewed. For 97% of the villages surveyed, the number of villagers interviewed was between 9 and 11 (the original aim of 10 villagers was achieved in 89% of the villages surveyed). Only one of the villages surveyed yielded fewer than five interviews.

#### Questionnaire design

In the analysis phase of the survey, the questionnaire design was evaluated with respect to consistency of responses, apparent ease of answering the questions and length of the questionnaire. These aspects of the design were assessed by comparing answers for selected sets of questions, assessing the range of responses for questions, asking field experts about the adequacy and plausibility of answers, and inspecting the magnitude and nature of missing data. Based on these assessments, the questionnaire was found to be quite robust.

#### Preparation

Post-survey meetings of the survey teams were undertaken by the regional and national coordinators to evaluate the conduct of the survey and interviews. Difficulties with implementation of the survey scheme were noted, such as access to some villages. Difficulties with data entry were also noted; some (n = 9) teams initially needed support with data entry in the Palmtops, with some having worked on paper for later transfer to digital format; we discouraged this strongly, but found out later than one team had continued to do so. For 24 pairs of villages teams had used similar Village Identification Numbers (IDs), but we were able to resolve these cases and assign all to unique IDs. Many of the surveyed villages had a mismatch between the GPS locality as measured by the teams in the field and the GPS locality from the original village database, but all of these were resolvable using the recorded GPS data. Some questions were identified as lacking tight or consistent definitions, for example in defining ‘around the village’ or ‘forest trip’. We gave careful consideration to the implications of these general statements for our inferences, and revised questions were documented for future surveys.

### (ii) Data acquisition

#### Respondent selection

As described above, respondents were chosen without letting them know that the survey was targeted towards orangutans. Nevertheless, potential voluntary response bias, or over-representation of respondents with strong opinions, was assessed by comparing the responses of those respondents who were randomly selected (64% of the sample) with those who were suggested (18%) and those who volunteered themselves (18%). There were no substantive differences between the three groups with respect to demographics, reported presence or relative encounters with orangutans, nor responses regarding threats, trends or attitudes to the forest.

#### Respondent reliability

Overall, 1169 interviews (16.7%) were assigned to the lowest reliability level, either because the respondent could not reliably identify an orangutan, or because all records within a village were identical for these questions. It is highly unlikely that 10 respondents in one village gave exactly the same responses (including the same spelling of local species names and responses to false positives). The latter therefore suggests that either the animal photos were not used, or that the questions had been asked to groups rather than single respondents. We conservatively decided to assign these interviews (n = 598) to the lowest level of reliability, though this involved some loss of potentially reliable respondents.

For the higher levels of reliability, 919 respondents (13.2% of all respondents) could identify an orangutan and at least one of the other two selected species, and were assigned to reliability level 2, and 4885 (70.1%) could identify all three selected species, and were assigned to reliability level 3. When categorized by ethnicity, immigrants had the largest proportion of respondents that could not reliably identify an orangutan (36.8%), followed by Malay, Banjar and Kutai people (15.2%), Dayaks (13.2%), and formerly nomadic peoples (Punan and Ut) (0%). As described above, respondents who were classified as medium or high reliability were included in the analyses regarding orangutans. Thus 83% of the 6972 respondents were included in these analyses. This group comprised over 90% of the respondents who reported seeing orangutans in the last year. In light of this, possible bias due to excluding unreliable responses was not considered to be a substantive issue.

Four approaches were taken to assess potential respondent bias. First, one of the survey questions documented the precision of responses regarding the location of reported sightings of orangutans on a scale of 1 (most precise, geographic coordinates provided), 2 (specific location), 3 (route to a named place), 4 (distance and bearing from a named point) and 5 (least precise, known geographic location or forest block). Among respondents who reported sightings, 86% were given a rating of 1–3. Similarly high figures were obtained when we considered only reliable respondents (86±1%). Secondly, analyses of variance were undertaken for questions that were more likely to have a common response among villagers (e.g., orangutan seen around the village in the last 12 months, someone in the village ever having killed an orangutan, etc). The results revealed significantly greater homogeneity of responses within villages compared to among villages. Third, conflicting temporal responses were examined by comparing individual responses to a range of overlapping questions with different timeframes. For example, less than 2% of those who reported ever seeing an orangutan gave conflicting responses to the various questions about seeing an orangutan in the last 12 months.

The fourth approach addressed the variation in villagers' responses, within villages, to questions about orangutans; it was acknowledged that the interpretation of these results was limited, since it was expected that even within the same village, respondents would have different propensities to encounter orangutans or simply differ randomly. Among reliable respondents, the same responses to the question, ‘have you ever seen an orangutan’, were documented for 19% of villages (in 6% of villages, all villagers responded positively; in 13% of villages, all villagers responded negatively). Confining attention to those villages in which any respondent reported having ever seen an orangutan, in 43% of villages a positive response was reported by more than a quarter of the villagers, and in 27% of villages a positive response was reported by more than half of the villagers. As anticipated, responses to the question ‘how many orangutans have you seen in the last year’ were more variable. In 41% of villages at least one respondent reported seeing one or more orangutans in the last year; sightings were reported by more than one person in 61% of these villages, and by more than a quarter of respondents in 14% of the villages. Responses about conflict and killing were also variable. For the question whether an orangutan had ever been killed in the village, it was common that if one person reported that no orangutan has been killed in the village, all other people in that village similarly reported that no orangutan had been killed. However, there was much less consistency for claims that orangutans had been killed in a village. Some of this was related to age and length of residence in the village, and it may also point to a lack of information flow within villages (i.e. the killing of an orangutan does not become known to everyone); alternatively, it may imply that killing is under or over-reported by individual respondents, possibly because killing orangutans is illegal.

### Analysis

#### Missing data

As described above, three datasets were derived. The dataset most informative for our analyses was dataset (ii) based on complete responses after inferring missing data where this was obvious and unambiguous, because this accounted for around 80% of all data for the variables considered to be most relevant in the analyses.

#### Statistical methods

The analysis proceeded as described above. Based on comparison of the model deviances, the multi-level models provided a superior fit to the data compared with analogous models that ignored the survey design (villagers nested within villages). The use of a multi-level model thus enabled us to adequately represent, rather than confound, the two sources of variation: variation among villages, and variation among villagers within a village [34].

#### Sensitivity analyses

As expected, fitting alternative statistical models to the same dataset resulted in differences in the size and significance of the various effects. However, in most cases these were consistent and interpretable. High levels of robustness were observed across models for inferences about factors associated with presence/absence of orangutans, perceived past and future trends in orangutan abundance, and attitudes to the forest. Some sensitivity was seen in the inferences on factors associated with relative encounter rates, conflict, and killing. Reassuringly, the substantive inferences about all of these outcomes were consistent across the five sub-sample analyses (where each sub-sample was a simple random sample of 80% of the villagers.).

#### Orangutan presence and threats

Population estimates of quantities of interest, such as relative abundance, hunting and human-orangutan conflict, were obtained by scaling up the survey results to the relevant population, using sampling weights based on the survey design. For this, we compared the demographics of the respondents with available 2006 and 2008 census data obtained from the Indonesian Bureau of Statistics for all residents in the three provinces in the sampling frame (East, West and Central Kalimantan). As anticipated, there were differences in the representation of gender and religion. For gender, 88.8% of respondents were male, compared with approximately 52% in the population. For religion, Muslims and Christians had approximately equal representation in the sample (although this differed by province: 47% Islam, 34% Christian in Central Kalimantan; 61%, 37% in East Kalimantan; 36%, 63.5% in West Kalimantan), while their representation in the census data was highly unequal (82% Muslim and 16% Christian for East Kalimantan in the 2006 census, and 64% and 36% for West Kalimantan in the 2008 census). Other religions accounted for less than 1% of the population. For ethnicity, the majority of respondents were classified as of Dayak origin (66.3%), followed by predominantly coastal Malay, Banjar and Kutai people (16.8%), immigrants (Javanese, Balinese, Buginese, etc., 16.5%), and formerly nomadic people (Punan, Orang Ut, 0.3%).

At the village level, the sampling weights reflected the number of high/medium/low threat villages in the sample compared with the number in the sampling frame. At the villager level, it was decided to apply equal weights to all respondents since the demographic distribution of the sample was considered to reflect the population that has propensity to encounter orangutans. Thus the target population is not the whole census population, but was taken to be the sub-population that matched the sample profile with respect to gender, religion and ethnic group.

It is worth noting that while the sample's non-representativeness with respect to the census population necessitates careful estimation of population values, by design the sample was regarded as representative of those villagers who are more likely to encounter orangutans; thus the over-representation of males and equal representation of the major religious groups in the sample was appropriate for the within-survey analyses, in particular understanding the factors associated with orangutan conservation.

Finally, a finite population correction factor [35], [36] was applied to account for the large sample (40% of the villages in the sampling frame). The uncertainty associated with these estimates was also calculated and expressed as confidence intervals. The large sample size in the survey (nearly 700 villages and 7000 villagers) resulted in quite precise sample statistics and thus reasonably accurate population estimates. As an illustration, based on these sample sizes, an estimated proportion (e.g., of presences) among villagers will have a standard deviation (s.d.) of at most 0.006; a corresponding estimated number of presences in the sample will have a s.d. of at most 40; and the estimated number of presences in the population will have a s.d. of at most 105. It is acknowledged that this does not take into account adjustments for misclassification, bias and other data quality issues, as these issues were addressed to some extent in the sensitivity analyses, as described above.

Our method for testing the accuracy of our ‘relative encounter’ measures in six village areas for which there was high quality and recent field survey information did not give results with sufficient resolution to determine the relationship between the RE levels and estimates of orangutan density from traditional line transect methods. Five village areas with relatively high orangutan densities were classed in the highest RE class based on interview results, while one village with very low orangutan densities was classed in the lowest RE class (Table 1). Although this match of extreme values is encouraging, the lack of intermediate densities makes it impossible to judge how well RE classes 2 and 3 would have correlated with orangutan densities. Future surveys should ensure that accuracy assessment includes areas with suspected low, medium, and high species densities.

**Table 1:** Orangutan densities versus relative encounter rate classes based on interviews.

	RE score from interviews	RE class	Density estimate from line transect surveys (95% CI between brackets)
Lesan village 1 (Muara Lesan)	0	1	0.13 (0.06–0.28)
Lesan village 2 (Lesan Dayak)	0.278	4	1.54 (1.16–2.03)
Kinabatangan village 1 (Gomantong)	0.100	4	1.11 (0.60–2.06) 2.91 (1.53–2.53)
Kinabatangan village 2 (Sukau)	0.231	4	3.14 (1.64–6.01) 2.15 (1.14–4.02)
Kinabatangan village 3 (Abai)	0.043	4	1.35 (0.72–2.50) 3.14 (1.64–6.01)
Kinabatangan village 4 (Bilit)	0.092	4	2.15 (1.14–4.02) 3.93 (2.03–7.64)

## Discussion

Surveys give an approximation of reality, and the most relevant question is whether that approximation provides reliable information to solve a particular problem. In addition, because of general resource constraints in conservation [37], [38], and the urgency of the problems we seek to solve, it is imperative that methods for gaining information are time and cost-effective. In this study we have systematically investigated representativeness, accuracy, relevance and effectiveness of interview-based surveys for orangutan conservation. We felt compelled to do this in considerable detail because there was and still is scepticism among conservation practitioners regarding the usefulness and accuracy of interview surveys. For example, colleagues working in Sierra Leone who were keen to test the same methods for surveying chimpanzees (*Pan troglodytes*) reported considerable reluctance among donors and fellow researchers to support interview based methods (H. Kühl, pers. comm. to EM). Survey design, data acquisition and analysis of interview surveys have not always fulfilled scientific requirements for rigour or robustness in conservation research. This may at least partially explain the generally negative perception of interview-based studies in conservation. Such perceptions might also be a cultural issue. Conservation scientists, who are mostly trained as ecologists, might be more inclined to apply field-based techniques involving ecological variables rather than social or management-focused ones [39], [40], [41].

Our analyses indicate that interview-based surveys, if well designed and carefully implemented in the field, can provide robust and cost-effective tools in the conservation of relatively easily recognized species [42]. The results of the present orangutan survey study provide what is probably the biggest leap in understanding of conservation status and needs for the orangutan's Kalimantan range of the last decade. The surveys have covered much more ground than previous survey efforts based on nest counts [17], and provided a clearer picture of orangutan encounter rates at least for some locations. Also, the surveys identified several orangutan populations that were previously unknown. More importantly, however, the surveys have clarified the spatial variation in threats to orangutans, in enabling factors such as public awareness about conservation laws, and in a range of socio-cultural data that help us to better understand ultimate drivers of orangutan population declines. The question is whether these findings are reliable and whether the interview surveys offer a cost and time-effective alternative to traditional survey methods.

Analyses of the study design (sampling frame, sampling extent, field methods and questionnaire) and data acquisition (respondent selection, respondent reliability, and respondent recall) indicate that the surveys were methodologically sound. Difficulties with data entry could have been addressed earlier and more effectively through better training of the survey teams, but overall we are satisfied with the data acquisition process. Our control mechanisms for judging the reliability of the respondents provided a useful way to test the sensitivity of the method to the quality of the information. The significant coverage (40%) of the orangutan range in Kalimantan, means that extrapolation from respondents to the wider population is far more valid for this survey than for previously applied methods.

One area for improvement in future interview surveys is to more effectively address the social-desirability bias. This is a systematic error caused by respondents providing dishonest answers in order to project a favourable image of themselves relative to prevailing social norms [43] [48]. This could be especially common when questions are asked about sensitive topics, like in the present case, the illegal hunting of orangutans. Randomised response techniques, like those recently trialled to assess illegal fly fishing in Wales [29] could be considered for inclusion in future orangutan interview surveys. Anonymous self-completion of questionnaires has also been shown to reduce social desirability bias in some contexts [47]. Another common approach is the application of a separate questionnaire to inform a social desirability scale which can then be used as an adjustment factor in the analyses [47], although these scales have commonly been constructed for populations in developed countries and have important drawbacks such as extending the interview time.

Another issue of concern is the extent to which interview teams could make up the data rather than obtain actual interviewee responses. Cheating and plagiarism cause problems in many areas of research [44], [45], and we are familiar with a range of examples from Indonesia in which field assistants were discovered to have been creating field data out of thin air. In this study, we tried to address cheating in a number of ways, although each has limitations. Firstly, we asked interview teams to take GPS labelled photographs of the villages so that we could determine whether the teams had actually been to the pre-selected village. This method did not prove to be very useful because we received tens of thousands of photos which, given current technology and our own programming abilities, were logistically hard to organize and effectively use as verification. We note, however, that simply requiring teams to submit such data may have reduced the probability of data fabrication. Secondly, we had hoped to be able to use the automatic date and time stamp that marks the start and finish of the interview to check whether interviewers had spent a realistic amount of time on the interviews. This information was available for only 4,894 of the 6,972 interviews, suggesting that either the date/time stamp was not functioning well, or that interview teams wrote down answers on paper first before entering them into the palm tops. This was also indicated by 176 interviews that were recorded to have taken less than 1 minute, an impossible amount of time to do an entire interview. Unfortunately, we cannot use this information to detect potential fraud, because there were obviously technical problems with data entry as well. Again, better training on data entry could have provided a more rigorous control mechanism. Thirdly, we used information on recall and consistency to evaluate consistency of responses within villages and to see whether there were obvious cases of illogical responses. Again, this does not tell us whether there was potential fraud in data entry by the interviewer, nor whether the interviewee was making up information. Finally, we undertook analyses of random subsamples of the data, which showed general consistency among subsamples in results and inferences. However, this does not eliminate the possibility of across-the-board cheating. Moreover, the choices about the number and size of the subsamples were arbitrary; more comprehensive sub-analyses may be required to identify cheating in this manner.

One approach to evaluation of cheating that we didn' t pursue here is to undertake a validation study. This is common in social surveys and entails re-interviewing a random sample of say 5-10% of villages (by different interviewers) and cross-checking responses with the original data [46], [47]. This was noted as a potential supplementary stage for future surveys.

The total budget for the survey was approximately US$ 221,000, which included the costs of hiring participating NGOs and a team of national and regional coordinators (US$ 162,000), supplies and equipment (US$ 27,000), training and workshops (US$ 19,000), and travel (US$ 13,000). If this is translated in expenses per unit area (excluding salaries) the cost per km^2^ would be US$ 2.00. To compare this cost with those of previous surveys to estimate presence or abundances of orangutans—bearing in mind that this study estimated four levels of relative encounter rates, and that this was one of many outcomes from the survey: line transect surveys in East Kalimantan for effective survey areas of about 100 km^2^, varied between US$ 1,000 and 1,700 (Nardiyono, pers. obs.), suggesting a cost of US$ 10–17/km^2^, which is similar to the estimated US$ 10/km^2^ estimated for a year-long line transect survey planned for a protected area in Sumatra (SW, pers. obs.). Helicopter surveys in Sabah, Malaysia, cost US$ 6–15/km^2^, with the proportion of land that is directly surveyed representing 8–16% of the total land area under investigation.

Obviously these different survey techniques have different purposes. Helicopter surveys can give accurate orangutan population estimates for large areas of flat or undulating forests or provide a cheap tool to check absence or presence in remote areas [48]. They are limited by the availability of helicopters, terrain, and weather conditions. Nest surveys either in transects or plots [8] are relatively time-consuming, but can provide accurate information about local orangutan densities and forest quality, especially in areas with limited spatial and temporal variation in nest decay rates [7]. However, they are rarely designed to provide information about direct and indirect threats and no information on social perceptions on conservation, or views on past and future changes.

Here we have shown that well designed interview surveys can provide relatively cheap information from large areas about the relative abundance of easily recognizable species such as orangutans and threats to their survival. Interview surveys are complementary to other techniques. We are not advocating any particular method over another, but would like conservationists to consider the potential benefits of interview surveys for surveying large, remote areas where little is known about the species. Before launching into any survey method, an assessment should consider the actual data needs, the envisaged survey outcomes, and how the results can be translated into improved conservation management. A recent example from Sierra Leone shows that these insights are now gaining traction in ape surveys [49], and we hope our findings will further encourage researchers and practitioners to explore interview-based methods.

## Supporting Information

Text S1 **Interview preamble and questionnaire details.**
(DOC)
