# Supplementary material for: Why Don't We Ask? A Complementary Method for Assessing the Status of Great Apes
Source: PLoS One. 2011 Mar 31;6(3):e18008. doi: 10.1371/journal.pone.0018008 (PMC3069039; doi:10.1371/journal.pone.0018008)
Supplement: Text S1 — Interview preamble and questionnaire details. (DOC) [file pone.0018008.s001.doc]

### **Supporting Information – Interview preamble and questionnaire details**

## Preamble

Before beginning the survey, the survey teams were asked to read the following statement exactly as written (translated from Indonesian)

"Thank you for giving me the opportunity to talk to you. My name is (insert name here). I am here to conduct a simple survey in your village. This is part of a large survey-based research project that is being conducted across all of Kalimantan. I am conducting this survey because I am interested in gathering information about animal presence and absence in the forests around your village, understanding the threats to wildlife in your area, and learning about your relationship with the forest.

I would like to ask you some basic questions. Before I do, I would like to let you know that you are not obliged to answer any of these questions, or to participate in any way. There is no punishment for not participating, and you will not be paid for your participation or compensated in any other way. If you agree to participate, you are free to stop at any point in the process. You may ask me questions at any time in this process.

I would also like you to know that this survey has nothing to do with law enforcement. You will not be punished for any answers you give or any information that you provide. We are simply interested in gathering accurate data. Your responses will be confidential. Your name will not be recorded, and results form this survey will only be reported in aggregate. Do you have any questions?"

## Village related questions in orangutan interview survey

Answers to these questions were entered on paper and later transferred to an data base.

After entering a village and finding the local village head or other local leader, the survey teams asked XX questions about the village and its people.

1. Name of the Subdistrict in which the village is located
2. Name of the village
3. GPS coordinates of village
4. Short descriptive history of the village
5. Year in which the village was established
6. Population size of village
7. Number of women
8. Number of men
9. Number of families
10. Main religious affiliations in percentages
11. Number of schools in the village
12. Number of grave yards in the village and whether these were Muslim or Christian
13. Description of the presence of customary forest land, claimed or managed by traditional law
14. Description of local folklore regarding orangutans
15. The presence of different agricultural production types or other sourced of income from natural resources in the villages, answered as yes/no: oil palm, coconut, rice, rubber, cacao, pepper, vegetables, hunting, traditional mining, fishing, and non-timber forest products.
16. The name of industrial companies (plantations, natural forest, mining) in the village area.

## Questionnaire design

The questions in Episurveyor can have either several different responses:

- (OPEN) a free text response
- (NUM, XX) a numerical response, and units
- (SINGLE: X, Y, Z) respondent must choose exactly one of provided choices
- (ANY: X, Y, Z) check all that apply

Subsequent to the introduction, the following questions were asked (in red are the questions in Indonesian.

**I. PART ONE: BASIC INFORMATION**

1. Location name (OPEN) Nama lokasi
2. Location type (SINGLE: village, logging camp, mining camp, other) – Tipe lokasi (Kampung, Kemp HPH, Kemp tambang, Lainnya)

If other, what? (OPEN) – Jika yang lain, apa?

1. GPS coordinates (NUM, UTM coordinates, geographically bounded) – Koordinat lokasi kampong (UTM)
2. Date (NUM, DATE) – Tanggal
3. Time (NUM, TIME) – Waktu
4. Interviewer (SINGLE: list interviewers' names, other) – Nama pewancara (daftar pewancara)

If other, who? (OPEN) – Jika lain, siapa?

1. How was the interviewee selected? (SINGLE: randomly, you sought them out following another’s suggestion, volunteered, other) – Bagaimana menyeleksi interview? (acak, saran orang lain, sukarela, lainnya)

If other, how? (OPEN) – Jika lain, bagaimana?

*Information gathered about interviewees*

1. Age (NUM, YRS) – Umur
2. Sex (SINGLE: FEMALE, MALE) – Jenis kelamin
3. Ethnic background (OPEN) – Suku
4. How many years have you lived here (OPEN) – Sudah berapa lama tinggal di sini?
5. Religion (SINGLE: Muslim, Christen, Hindu, Buddhist, other) – Agama (Islam, Kristen, lainnya)

If other, what? (OPEN) – Jika lainnya, apa?

1. How often do you go into the forest? (SINGLE: 5 or more times a week, 2-4 times a week, 1-2 times a week, 1-2 times a month, 1-2 times a year, never) – Seberapa sering anda masuk ke hutan? (5 kali atau lebih dalam seminggu, 2- 4 kali seminggu, 1- 2 kali seminggu, 1-2 kali sebulan, 1- 2 kali setahun, tidak pernah)

If answer ≠ never, Why do you go to the forest (SINGLE: Logging, hunting, mining, collecting non-timber forest products, other) – Jika jawaban tidak sama dengan tidak pernah, apa alas an utama anda pergi ke hutan? (menebang pohon, berburu, menambang, mencari hasil hutan non kayu, lainnya)

If other, what? (OPEN) – Jika lainnya, apa?

If answer ≠ never, when you go to the forest, do you spend the night there? (yes, no) – Jika jawaban tidak sama dengan tidak pernah, jika pergi ke hutan, apakah anda bermalam?

If = yes, how many nights per trip? – Jika ya, berapa malam setiap kali ke hutan?

If answer ≠ never, What time of day do you typically go to the forest? (ANY: morning, daytime, evening, night) – Jika jawaban tidak sama dengan tidak pernah, jam berapa anda biasanya pergi ke hutan (pagi, siang, sore, malam)

If answer ≠ never, When was the last time you went to the forest (yesterday, within the last 7 days, within the last 30 days, within the last 6 months, within the last year) – Jika jawaban tidak sama dengan tidak pernah, kapan anda terakhir kali pergi ke hutan (kemarin, dalam satu minggu ini, dalam satu bulan ini, dalam setengah tahun ini, dalam satu tahun ini)

1. Are there any rambutan (=*Nephelium lappaceum*) or durian (=*Durio* spp.) trees in the village (yes/no) – Apakah ada pohon rambutan dan durian di kampong ini (ya/tidak).
2. How long ago did rambutan/durian most recently fruit ? (NUM: MOS, if there are no rabutan/durian trees in the village write " don’t know") Write 0 if a fruit season is going on – Kapan pohon durian dan rambutan terakhir kali berbuah? (ANGKA: bulan, tidak ada rambutan/durian di kampung ini, tidak tahu), tulis 0 jika sedang musim buah.

**II. PART TWO: ASSESSMENT OF INTERVIEWEE RELIABILITY**

To assess the interviewees' knowledge of local fauna, we showed them full body images of a range of species, such of which do not occur in Borneo: 1. Pangolin (*Manis javanica*), 2. Banteng (*Bos javanicus*), 3. Proboscis Monkey (*Nasalis larvatus*), 4. Red Leaf Monkey (*Presbytis rubicunda*), 5. Gibbon (*Hylobates* sp.), 6. Douc Langur (*Pygathrix nemaeus*, a species not occurring on Borneo), 7. Sumatran Elephant (*Elephas maximus*, a species not occurring in the Indonesian part of Borneo), 8. Red Bird of Paradise (*Paradisaea rubra*, a species not occurring on Borneo, but known from one of Indonesia’s banknotes), and 9 . Bornean Orangutan (*Pongo pygmaeus*).

Show photograph or picture of each, and for each ask the local name of the species. – Perlihatkan poster satwa dan tanyakan nama local dari masing-masing satwa tersebut.

1. What is the (local) name of this animal? – Apa nama daerah untuk binatang ini? (OPEN)
2. Have you ever seen this animal in the forest? (SINGLE: yes, no) – Apakah anda pernah melihat binatang ini di hutan? (Ya, tidak)

**III. PART THREE: ORANGUTANS**

1. Have you ever seen an orangutan in your village area? (SINGLE: yes, no, not sure) – Pernahkah anda melihat orangutan di sekitar kampung ini? (ya, tidak, tidak yakin)

If yes, how many orangutans have you seen in the last year? (NUM) – Jika ya, berapa ekor orangutan yang anda lihat selama satu tahun terakhir?

If yes, where do you usually see orangutans? (SINGLE: forest, gardens or fields, along the road, other) – Jika ya, dimana biasanya anda melihat orangutan? (hutan, kebun atau ladang, jalan, lainnya)

If other, where? (OPEN) – Jika lain, dimana?

If yes, when did you last see an orangutan? (SINGLE: within last week, within last month, within last year, more than a year ago) – Jika ya, kapan anda terakhir kali melihat orangutan? (Dalam satu minggu ini, dalam satu bulan ini, dalam satu tahun ini, lebih dari setahun yang lalu)

If yes, was it a mother with a baby? (SINGLE: yes, no, not sure) – Jika ya, apakah induk dengan anaknya? (Ya, tidak, tidak tahu)

If yes, in what type of location did you last see an orangutan? (SINGLE: forest, gardens or fields, along the road, other) – Jika ya, di lokasi seperti apa anda terakhir kali melihat orangután? (hutan, kebun atau ladang, jalan, lainnya)

If other, where? (OPEN) – Jika lainnya, dimana?

If yes, what was the location where you last saw an orangutan? (OPEN) – Jika ya, dilokasi mana terakhir kali anda melihat orangután?

If yes, what is the precision of the observation? (SINGLE: 1, 2, 3, 4, 5; where) – Jika ya, seberapa besar tingkat keyakinan akan posisi tersebut?

1 most precise, geographic coordinates provided for sighting location – Lebih detil koordinat geografi lokasi dimana dilihat

2 sighting assigned to a specific location (e.g., Mr Erik’s garden) – Lokasi yang spesifik dimana melihat (contoh kebun Pak Erik)

3 sighting described as a route from a named place (e.g., Sungai Lesan, 5 km from main camp on the North side of the river) – Memberikan gambaran yang jelas dari salah satu kawasan (contoh, Sungai Lesan, 5 km dari kemp induk di sebelah utara sungai)

4 sighting described as a distance and bearing from named point (e.g., 10 km North East from Samarinda) – Memeberikan gambaran yang jelas jarak dan arah dari suatu lokasi (contoh, 10 km dari Samarinda Utara Timur)

5 least precise, sighting described as being in a known geographic region or forest block (e.g., in Gunung Gajah concession or along the Mahakam river) – Sedikitnya ada gambaran dimana dilihat serta informasi geografi secara regional disuatu kawasan hutan (contoh, di HPH Gunung Gajah atau disepanjang sungai Mahakam)

1. Have you ever seen an orangutan anywhere else? If yes, where (OPEN) – Pernahkah anda melihat orangutan di tempat lain? Jika ya, dimana?
2. Do orangutans ever come into your gardens or fruit trees? (SINGLE: yes, no, don’t know) – Apakah orangutan datang ke kebun anda atau pohon buah anda? (ya, tidak, tidak tahu)

If yes, how do you know? (SINGLE: I have seen them, other people have seen them, I found traces of them feeding, I saw its nest) – Jika ya bagaimana anda tahu? (melihat sendiri, orang lain yang melihat, melihat bekas makan, melihat sarang)

If yes, how often? (SINGLE: weekly, monthly, once a year, very rarely) – Jika ya, seberapa sering? (setiap minggu, setiap bulan, sekali setahun, sangat jarang)

If yes, what foods to they prefer to eat in your gardens? (OPEN) – Jika ya, makanan apa yang mereka sukai di kebun anda?

If yes, what is your reaction? (SINGLE: kill them, attempt (unsuccessfully) to kill them, try to scare them, ignore them, other) – Jika ya, apa reaksi anda? (membunuhnya, mencoba membunuhnya, menakutinya, mengusirnya, lain)

If other, what do you do? (OPEN) – Jika lain, apa yang anda lakukan?

1. When was the last time that someone in your village killed an orangutan? (SINGLE, within the last week, within the last month, within the last year, within the last 5 years) – Kapankah orang terakhir kali membunuh orangutan di kampung ini? (dalam satu minggu ini, dalam satu bulan ini, dalam satu tahun ini, dalam 5 tahun ini).

If yes, how many orangutans have been killed in the village area in the last year (NUM, INDIV) – Jikaya, berapa orangutan yang dibunuh dalam satu tahun terakhir di tempat ini?

Why were orangutans killed? (ANY: traditional medicine, food, to sell babies, because they were crop raiding, hobby/sport, because I was afraid/self-defense, I was paid to kill it, don’t know, other) – Kenapa (obat tradisional, untuk dimakan, jual anaknya, hama, hobi/olahraga berburu, karena takut/membela diri, dibayar untuk membunuh, tidak tahu, lainnya)

If other, why? (OPEN) – Jika lainnya, kenapa?

Are there specific people in your village who are specialized orangutan hunters? (yes, no, don’t know) – Apakah ada orang yang khusus berburu orangutan di kampung ini? (ya, tidak, tidak tahu)

1. How many orangutans have you killed in your life? – Berapa jumlah orangutan yang pernah anda bunuh?

If ≠ 0, why did you kill it/them? (ANY: traditional medicine, food, to sell babies, because they were crop raiding, hobby/sport, because I was afraid/self-defense, I was paid to kill it, don’t know, other) – Kenapa anda membunuh orangutan? (obat tradisional, untuk dimakan, jual anaknya, hama, hobi/olahraga berburu, karena takut/membela diri, dibayar untuk membunuh, tidak tahu, lainnya)

If other, why? (OPEN) – Jika lainnya, kenapa?

1. How many orangutans live in your area, compared to ten years ago? (SINGLE: more than ten years ago, about the same, fewer than ten years ago, don’t know) – Berapa jumlah orangutan yang ada diwilayah anda, bandingkan dengan 10 tahun yang lalu (lebih banyak dibanding dari10 tahun yang lalu, kira-kira sama, lebih sedikit dari 10 th yang lalu, tidak tahu)

Why? (OPEN) – Kenapa?

1. How many orangutans do you think will be here in ten years time? (SINGLE: more than now, about the same, fewer than now, none, don’t know) – Menurut anda berapa orangutan yang ada disini dalam 10 tahun kedepan? (lebih dari sekarang, kira-kira sama, kurang dari sekarang, tidak ada, tidak tahu)

Why? (OPEN) – Kenapa?

1. What, in your opinion, could cause orangutan populations to decrease in this area? (ANY: hunting, logging, mining, oil palm, fires, other, orangutans are not threatened here, other) – Apakah penyebab berkurangnya orangutan di daerah ini? (Perburuan, penebangan hutan, penambangan, kebun kelapa sawit, kebakaran hutan, lainnya, orangutan disini tidak terancam, lainnya)

If other, what? (OPEN) – Jika lain, apa?

1. Are orangutans protected by local customs or rites? (SINGLE: yes, no, don’t know) – Apakah orang-utan dilindungi oleh adat? (ya, tidak, tidak tahu)
2. Do you think orangutans should be protected by local customs or rights? (SINGLE: yes, no, don’t know) – Menurut anda, apakah orang-utan perlu dilindungi oleh hukum adat (ya, tidak, tidak tahu)

Why do you think this? (OPEN) – Kenapa?

1. Are orangutans protected by Indonesia law? (SINGLE: yes, no, don’t know) – Apakah orang-utan dilindungi oleh hukum Indonesia? (ya. Tidak, tidak tahu)

Why? (OPEN) – Kenapa?

1. Do you think orangutans should be protected by Indonesian law? (SINGLE: yes, no, don’t know) – Apakah orang-utan harus dilindungi oleh hukum Indonesia? (ya, tidak, tidak tahu)

**IV. PART THREE: FOREST USE AND MANAGEMENT**

1. Do forests hold significant cultural or spiritual significance for you and your family? (SINGLE: very significant, somewhat significant, not significant, don’t know) – Apakah hutan memegang peranan dalam budaya dan spiritual yang cukup signifikan bagi anda dan keluarga anda? (sangat significan, cukup significan, tidak significan, tidak tahu)
2. What economic benefits do you receive from the forest? (SINGLE: timber, rattan, gaharu, honey, mining, hunting, traditional medicine for sale, other) – Keuntungan ekonomis apa yang anda dapatkan dari hutan? (kayu, rotan, gaharu, madu, tambang, binatang buruan, obat tradisional, lainnya)

If other, what? – Jika lainnya, apa?

1. How important do you think forests are for your family’s health? (SINGLE: very important, somewhat important, not important, don’t know) – Menurut anda seberapa penting hutan untuk kesehatan anda dan keluarga anda? (sangat penting, cukup penting, tidak penting, tidak tahu)

Why? (OPEN) – Kenapa?

1. Do you think that conversion of natural forest is good for you and your family? (SINGLE: very beneficial, somewhat beneficial, not beneficial, don’t know) – Apakah pembukaan hutan akan memberi keuntungan bagi anda dan keluarga anda? (ya, tidak, tidak tahu)

If ≠ don’t know, why? – Jika tidak sama dengan tidak tahu, kenapa

1. End time.
